# Construction of prognostic nomogram based on the SEER database for esophageal cancer patients

**DOI:** 10.1016/j.clinsp.2024.100433

**Published:** 2024-07-29

**Authors:** Xiying Cao, Bingqun Wu, Shaoming Guo, Weixiang Zhong, Zuxiong Zhang, Hui Li

**Affiliations:** a Department of Thoracic Surgery, Beijing Chaoyang Hospital, Capital Medical University, Beijing, China; b Department of Thoracic Surgery, The First Affiliated Hospital of Gannan Medical University, Ganzhou City, Jiangxi Province, China; c Department of Thoracic Surgery, Huaxin Hospital, First Hospital of Tsinghua University, Beijing, China

**Keywords:** Esophageal cancer, Prognostic nomogram, Overall survival (OS)

## Abstract

Currently, the incidence of esophageal cancer continues to rise around the world. Because of its good early prognosis, it is of great significance to establish an effective model for predicting the survival of EC patients. The purpose of this study was to predict survival after diagnosis in Esophageal Cancer (EC) patients by constructing a valid clinical nomogram. In this study, 5037 EC patient samples diagnosed from 2010 to 2015 were screened by accessing the SEER database, and 8 independent prognostic factors were screened by various methods, and Cox multivariate regression was included to construct a prognostic model and nomogram for esophageal cancer. to estimate esophageal cancer recurrence and overall survival. Calibration of the nomogram predicted probabilities of 1-year, 3-year and 5-year survival probability, which were closely related to actual survival. In conclusion, this study validated that the column-line graphical model can be considered an individualized quantitative tool for predicting the prognosis of patients with EC in order to assist clinicians in making therapeutic decisions.

## Highlight

• The nomogram can help clinicians make treatment recommendations based on patient survival.
• This nomogram shows superior survival prediction ability.
• This nomogram provides guidance for the prognosis assessment of EC patients in terms of individualized tumor-specific survival prediction.
• Calibration and DCA curves were used to verify the consistency of the predicted survival rate with the actual results.

## Introduction

Esophageal Cancer (EC) is a major global health problem, and its incidence is increasing rapidly around the world.^1^^,^^2^ EC is a malignant tumor originating from the esophagus, which typically presents with progressive dysphagia.^3^^,^^4^ EC occurs mostly in middle-aged and elderly men, and its early treatment has a better prognosis and a higher 5-year survival rate after comprehensive treatment.^5^^,^^6^ A successful and accurate prognostic model for EC patients is therefore vital to their treatment, but a suitable model for predicting their survival is still lacking.

Currently, the gold standard for evaluating tumor prognosis is still the TNM classification.^7^^,^^8^ However, the TNM system has several disadvantages: Heterogeneity in disease stages is introduced by patients with similar TNM classifications, but different survival outcomes;^9^^,^^10^ the TNM classification cannot treat tumors, lymph nodes, or metastases as a continuous variable, and generally, if the TNM classification is too high, the prognosis will be worse.^11^^,^^12^ TNM does not incorporate variables other than genetics, mitotic rate, and histology that affect prognosis, resulting in poor outcomes in general.^13^^,^^14^ In addition, the American Joint Committee on Cancer (AJCC) seventh edition (2010) is widely used in the prognosis evaluation and clinical treatment of embassy cancer patients.^15^ However, due to the lack of demographic data, the application of this system is limited.^16^^,^^17^ Given the limitations of the TNM classification and the AJCC cancer staging manual, nomograms serve as a simpler and more advanced approach to assessing individual risk based on patient and disease characteristics. A nomogram is a visualization of a complex statistical model. In this method, multiple predictors are integrated, and then scaled line segments are drawn on the same plane according to a certain proportion as part of the regression analysis.^18^^,^^19^ The fundamental concept behind the nomogram involves developing a multivariate regression model, which includes widely used models like Cox regression and Logistic regression. Scores are assigned to each value level of the influencing factors in the model, based on their impact on the outcome variable (the magnitude of the regression coefficient), and summed up to calculate an overall score. Ultimately, the forecasted value of a specific outcome event is determined by the functional transformation linking the overall score to the likelihood of that event occurring. Every variable is individually enumerated, with each sub-variable being quantified into distinct points. Subsequently, the aggregate scores (Total points) of each variable are compared with the outcome scale to derive the forecasted probabilities.

Oncology can benefit from the nomogram in many ways: preoperative nomogram can assess surgical margins and risk of lymph node metastasis to help clinicians identify those who may benefit more from a larger surgery benefit patients.^20^^,^^21^ In short, nomograms may assist patients and physicians in making decisions about estimating tumor recurrence, tumor-specific survival, overall survival, benefit of adjuvant therapy, and treatment impact on quality of life.^22^, ^23^, ^24^ Therefore, this paper used Cox multivariate regression and nomogram to construct a prognostic model of EC.

### Materials selection and methods

This study received an access research license from the Surveillance, Epidemiology and End Results (SEER) database. According to clinical consensus, demographic characteristics (including age, gender, race) and clinicopathological factors involving tumor size, tumor grade, tumor stage (Summary stage, AJCC Stage), tumor size and regional lymph node involvement, and distant metastasis were analyzed as influencing factors.^25^, ^26^, ^27^, ^28^, ^29^, ^30^ The sample data of 7246 EC patients from 2004 to 2015 were determined by the software SEER*Stat 8.4.0.1. In this study, the AJCC staging criteria (Derived AJCC Stage Group, 7^th^ ed.) were used to classify EC patients according to different stages, excluding samples with unknown diagnosis, multiple occurrences, unknown gender and empty Derived AJCC Stage Group. Since the seventh edition of the AJCC standard was released in 2010, a total of 5049 samples were screened from 2010 to 2015, and the data with NA was filtered out, leaving 5037 samples in the end. To reduce the possible bias caused by the dataset from only one data center, multi-center or national databases were used, so this paper intended to include the clinical information of EC patients in the 2010‒2015 TCGA database for external validation. However, for the information on EC patients, the data range on TCGA was only up to 2013, and the data volume was only 180 rows. In order to ensure the integrity of the data set, this study used the bootstrap sampling method, and the esophageal patient samples obtained from the SEER database were trained according to the training method. The ratio of set to validation set sample size was 7:3 and allocated to the training cohort and the internal validation cohort. This study follows the STROBE statement.

R 4.0 was used for survival analysis, python 3.8 was used for data processing and univariate analysis, as well as three methods were used to screen variables. Initially, the univariate Cox model set a threshold of *p* < 0.01 for screening variables. Subsequently, a comprehensive subset regression analysis was employed to modify the peak value of R2, aiming to identify the optimal variable mix. Ultimately, Lasso regression combined with cross-validation was applied to identify the pairing of variables with the respective λ value at the point where the Mean Square Error (MSE) was at its lowest. In the multivariate Cox regression, the variables evaluated by the trio of methods were incorporated, followed by a stepwise backward regression to identify those with the lowest AIC value that the three methods ultimately screened. Models developed using these three techniques rely on the ROC curve, selecting the one with the highest AUC for the nomogram's construction. Ultimately, to confirm the model's calibration, the calibration curve's C-index was established, and Decision Curve Analysis (DCA) assessed the nomogram's clinical applicability.

## Results

### Patients’ characteristics

A total of 5037 EC samples diagnosed in 2010‒2015 were included, of which 3525 were assigned to the training set and 1512 to the validation set. In the entire cohort, the 1-year, 3-year, and 5-year EC-specific mortality rates were 55.3 %, 77.5 %, and 86.6 %, respectively. The demographic and tumor characteristics of the patients are shown in Table 1. Particularly, age, sex, and race, corresponding to age, sex, and race in the demographic characteristics, respectively; Derived_AJCC_Stage, Summary_stage, Histology_record_groupings, grade, tumor_size_group, respectively correspond to the tumor stage (Summary stage, AJCC Stage), regional lymph node involvement, and distant metastasis, tumor grade and tumor size in clinicopathological factors.

**Table 1:** Demographics and tumor characteristics of EC patients.

		All cases		Training		Validation		All cases		
Variables		N (5037)	%	N (3525)	%	N (1512)	%	1-year death rate	3-years death rate	5-years death rate
Age group	< 35	35	0.006948581	21	0.004169148	14	0.002779432	0.00394124	0.006398771	0.006419074
	35‒59	1323	0.262656343	912	0.181060155	411	0.081596188	0.243998567	0.253903251	0.254699679
	60‒75	2480	0.492356561	1759	0.349215803	721	0.143140758	0.457183805	0.474788841	0.4825768
	> 75	1199	0.238038515	833	0.165376216	366	0.072662299	0.294876388	0.264909137	0.256304448
Sex	Female	994	0.197339686	690	0.136986301	304	0.060353385	0.20064493	0.194266701	0.195552499
	Male	4043	0.802660314	2835	0.562835021	1208	0.239825293	0.79935507	0.805733299	0.804447501
Race	Black	314	0.062338694	213	0.042287076	101	0.020051618	0.069867431	0.069362682	0.066712517
	Other	392	0.077824102	275	0.05459599	117	0.023228112	0.088498746	0.085487586	0.081613939
	White	4331	0.859837205	3037	0.602938257	1294	0.256898948	0.841633823	0.845149731	0.851673544
Grade	Grade I	188	0.037323804	127	0.025213421	61	0.012110383	0.025080616	0.025595086	0.031866116
	Grade II	1609	0.319436172	1129	0.224141354	480	0.095294818	0.288068793	0.300998208	0.307427785
	Grade III	2092	0.415326583	1466	0.291046258	626	0.124280326	0.456825511	0.451753263	0.438560293
	Grade IV	68	0.013500099	46	0.00913242	22	0.004367679	0.013973486	0.014077297	0.013296653
	Unknown	1080	0.214413341	757	0.15028787	323	0.064125472	0.216051594	0.207576145	0.208849152
Summary stage	Distant	2158	0.428429621	1524	0.302561048	634	0.125868573	0.570763167	0.512669567	0.479825768
	Localized	843	0.167361525	586	0.116339091	257	0.051022434	0.09673952	0.109035065	0.12952774
	Regional	1659	0.329362716	1155	0.229303157	504	0.100059559	0.235041204	0.293831584	0.31132508
	Unknown/ Unstaged	377	0.074846139	260	0.051618027	117	0.023228112	0.097456109	0.084463783	0.079321412
Histology recorde groupings	8000‒8009: Unspecified neoplasms	73	0.014492754	44	0.008735358	29	0.005757395	0.022572555	0.017660609	0.016276937
	8010‒8049: Epithelial neoplasms, NOS	176	0.034941433	125	0.024816359	51	0.010125074	0.04120387	0.03583312	0.034387895
	8050‒8089: Squamous cell neoplasms	1393	0.276553504	981	0.194758785	412	0.081794719	0.311357936	0.291783977	0.284273269
	8140‒8389: Adenomas and adenocarcinomas	3154	0.626166369	2210	0.438753226	944	0.187413143	0.576137585	0.606859483	0.617148097
	8440‒8499: Cystic, mucinous and serous neoplasms	195	0.03871352	130	0.025809013	65	0.012904507	0.040487281	0.039160481	0.039889959
	8560‒8579: Complex epithelial neoplasms	37	0.007345642	29	0.005757395	8	0.001588247	0.008240774	0.008190427	0.007336084
Derived AJCC Stage	IA	397	0.078816756	271	0.053801866	126	0.02501489	0.032604801	0.036089071	0.050206327
	IB	290	0.057573953	198	0.039309113	92	0.01826484	0.038337513	0.043767597	0.049977075
	IIA	126	0.02501489	93	0.018463371	33	0.006551519	0.017556431	0.022267725	0.022696011
	IIB	617	0.122493548	440	0.087353583	177	0.035139964	0.078108205	0.102124392	0.111187529
	IIIA	756	0.150089339	527	0.104625769	229	0.04546357	0.104621999	0.134118249	0.143053645
	IIIB	233	0.046257693	161	0.03196347	72	0.014294223	0.034037979	0.045047351	0.046538285
	IIIC	298	0.0591622	208	0.041294421	90	0.017867778	0.063059835	0.064243665	0.063273728
	IIINOS	18	0.003573556	12	0.00238237	6	0.001191185	0.004657829	0.004351165	0.003897295
	IV	1739	0.345245186	1226	0.243398849	513	0.101846337	0.491580079	0.425902227	0.393168271
	UNK Stage	560	0.111177288	386	0.076632916	174	0.034544372	0.135435328	0.122088559	0.115772581
Tumor size group	< 42	1271	0.252332738	862	0.171133611	409	0.081199126	0.173056252	0.206296391	0.224667584
	43‒80	1323	0.262656343	924	0.183442525	399	0.079213818	0.27122895	0.275403123	0.272122879
	> 80	2443	0.485010919	1739	0.345245186	704	0.139765734	0.555714798	0.518300486	0.503209537

### Screening for independent prognostic factors

**Fig. 1:**
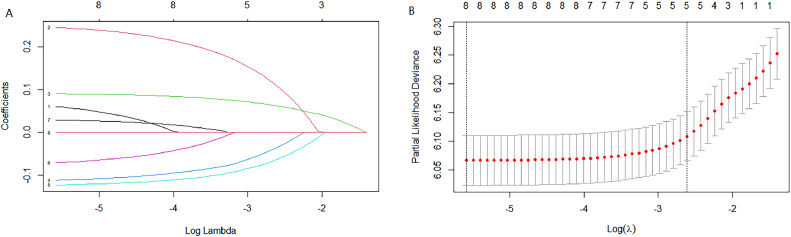
LASSO regression. (A) is a graph of lambda and regression coefficients when using LASSO regression + cross-validation to select variables; (B) is a graph of using cross-validation to determine the best lambda, when the mean square error MSE is the smallest, lambda is less than -5.

**Fig. 2:**
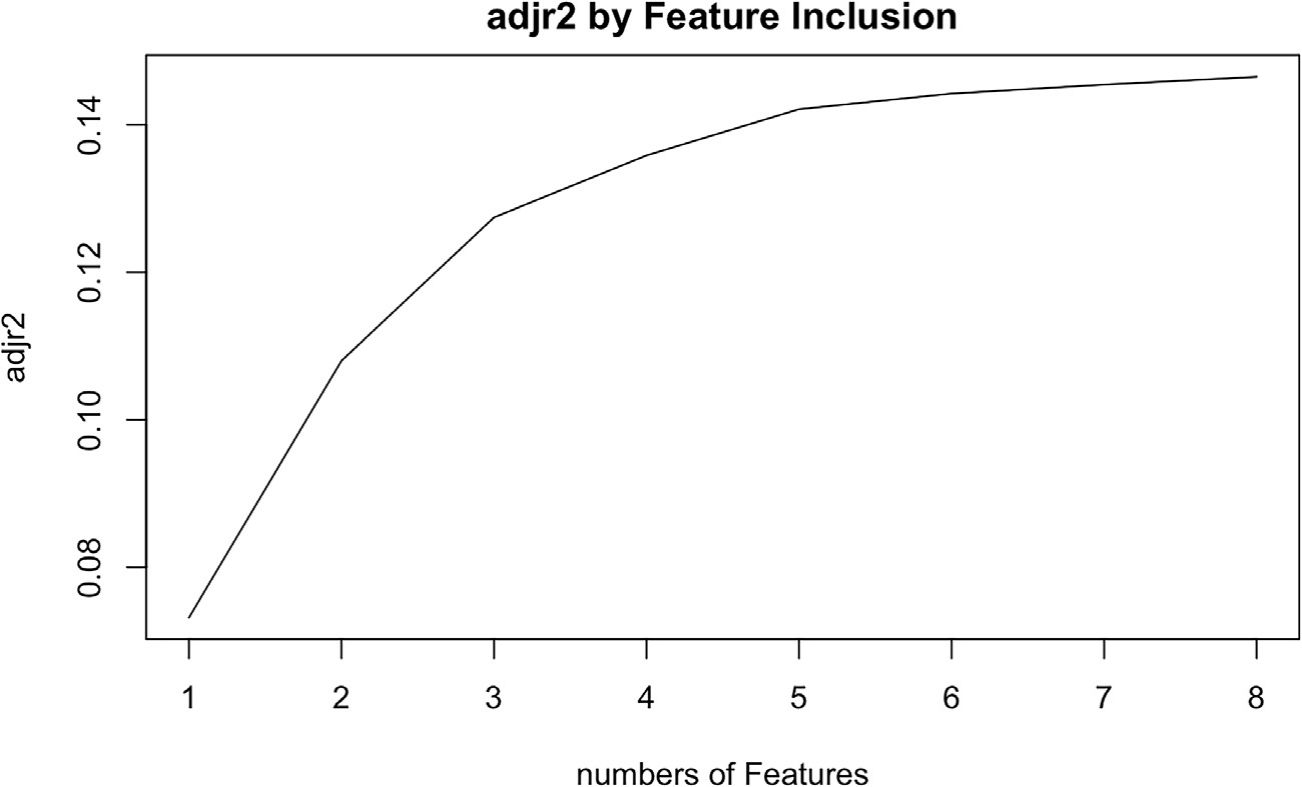
Adjr2 score by best subset features.

Cox univariate analysis was used to screen for variables with *p* < 0.01. Based on the univariate regression results, A multivariate Cox regression model was built based on variables with significant differences, and then variables with significant differences were included in the model. Backward stepwise regression was used, starting with all 8 predictors and performing backward regressions, removing one variable at a time, until it degraded the quality of the model. Excluding the feature variable with p-value > 0.01 and the largest each time, the p-value of each variable in the above model was obtained, as shown in Table 2. According to the p-value in Table 2, excluding race, the authors gathered 7 variables. Subsequently, LASSO regression and cross-validation were used to screen variables, which were used to improve the effect of model fitting and solve the problem of overfitting caused by multicollinearity. In LASSO regression, variables were selected and regularized while fitting a generalized linear model. Thus, LASSO regression can be applied to models and predictions regardless of whether the target variable is continuous, binary, or multivariate discrete, as shown in Fig. 1. A is a plot of lambda and regression coefficients when variables are selected. B uses cross-validation to determine the optimal λ value, which plots the cross-validation curve (red dotted line) and the λ series (error bars) along the upper and lower standard deviation curves. The two special value λ sequences along the line are represented by vertical dashed lines. A value of lambda.min indicates that a maximum cross-validation error has been achieved, while lambda.1se indicates the most regularized model with the least cross-validation error. According to the results in Fig. 1, lambda min gave 8 variables, lambda.1se also gave 8 variables, and it was determined to use 8 variables to build the model. Meantime, in order to overcome the controversy that the stepwise regression method cannot guarantee that the obtained model is the best model, this study adopted the best subsets regression (Best Subsets Regression, BSR), Adjr2 (adjusted R²) to judge the pros and cons of the model, and the model with the largest R² value was used in this study. The model constructed by the combination of variables was optimal. As shown in Fig. 2, 8 variables were included for model building. So far, univariate Cox regression has screened 7 variables with *p* < 0.01: age + gender + Derived_AJCC_Stage + Summary_stage + Histology_recode_groupings + grade + tumor_size_group; Optimal subset regression has screened 8 variables according to the adjusted R² maximum value: age + gender + race + Derived_AJCC_Stage + Summary_stage + Histology_recode_groupings + grade + tumor_size_group; LASSO regression + cross-validation using tuning coefficient = lambda.1se gave a model with good performance, and also gave a model with 8 variables: age + gender + race + Derived_AJCC_Stage + Summary_stage + Histology_recode_groupings + grade + tumor_size_group. It was found that based on the research results, the variables screened by optimal set regression and LASSO regression were the same. The variable combinations screened by the three methods were included in the multi-factor Cox model respectively, and the final model of the three methods was determined with the minimum AIC value using the stepwise backward regression method. Finally, draw three final model ROC curves and evaluate the best model with AUC value.

**Table 2:** Selected variables by multivariate cox proportional hazards regression analysis and prognostic score.

Variables	Chisq	df	p-value
Sex	8.437	1	0.0037
Derived AJCC Stage num	18.111	1	2.10E-05
Histology recode groupings num	25.478	1	4.50E-07
Summary stage	75.451	3	2.90E-16
Age group	25.995	3	9.60E-06
Race	0.316	2	0.8537
Grade	28.715	4	8.90E-06
Tumor size	22.153	1	2.50E-06
GLOBAL	129.97	16	<2.00E-16

Firstly, the variables screened by univariate cox were subjected to multi-factor Cox, and after the stepwise backward regression method, 6 characteristic variables were finally left, as shown in Table 3. The calculated AIC value was 43695.41. The variables screened by full Subset Regression (BSR) and LASSO regression were respectively subjected to Cox, and there were still 8 characteristic variables left in the stepwise backward regression method, as shown in Table 4. The AIC values were both 43691.46.

**Table 3:** Variables selection for Cox by backward elimination.

	Coef	S.E.	Wald Z	Pr(>|Z|)
**Derived_AJCC_Stage_num**	0.1013	0.012	8.46	<0.0001
**Histology_recode_groupings_num**	−0.0943	0.0157	−6.01	<0.0001
**Summary_stage = Localized**	−0.5445	0.1075	−5.07	<0.0001
**Summary_stage = Regional**	−0.5633	0.0545	−10.34	<0.0001
**Summary_stage = Unknown/unstaged**	0.6931	0.1421	4.88	<0.0001
**Age_group = Old**	0.1086	0.0452	2.4	0.0163
**Age_group = Senior**	0.6154	0.0523	11.77	<0.0001
**Age_group = Young**	−0.554	0.2611	−2.12	0.0338
**Grade = Grade II**	0.1716	0.1068	1.61	0.1083
**Grade = Grade III**	0.3665	0.1056	3.47	0.0005
**Grade = Grade IV**	0.336	0.1903	1.77	0.0775
**Grade = Unknown**	0.1763	0.1093	1.61	0.1066
**Tumor_size**	0.0002	0	4.45	<0.0001

**Table 4:** Variables selection for BSR/LASSO by backward elimination.

	Coef	S.E.	Wald Z	Pr(> |Z|)
**Sex = Male**	0.0719	0.0473	1.52	0.1288
**Derived_AJCC_Stage_num**	0.1013	0.012	8.46	< 0.0001
**Histology_recode_groupings_num**	−0.0882	0.0164	−5.38	< 0.0001
**Summary_stage = Localized**	−0.5374	0.1074	−5	< 0.0001
**Summary_stage = Regional**	−0.5606	0.0545	−10.29	< 0.0001
**Summary_stage = Unknown/Unstaged**	0.6992	0.142	4.92	< 0.0001
**Age_group = Old**	0.1147	0.0452	2.54	0.0112
**Age_group = Senior**	0.6379	0.0529	12.06	< 0.0001
**Age_group = Young**	−0.5525	0.2611	−2.12	0.0344
**Grade = Grade II**	0.1741	0.1068	1.63	0.1032
**Grade = Grade III**	0.3688	0.1057	3.49	0.0005
**Grade = Grade IV**	0.3517	0.1905	1.85	0.0648
**Grade = Unknown**	0.1766	0.1093	1.62	0.1061
**Race = Other**	−0.1489	0.0977	−1.52	0.1275
**Race = White**	−0.2163	0.0754	−2.87	0.0041
**Tumor_size**	0.0002	0	4.47	< 0.0001

In the multivariate models of the above three methods, only the variables selected by the single factor Cox were eliminated. Considering the principle that data fitting was encouraged but overfitting should be avoided as much as possible, a model with a smaller AIC value (BSR/LASSO) should be selected, that is, a combination of 8 characteristic variables.

**Fig. 3:**
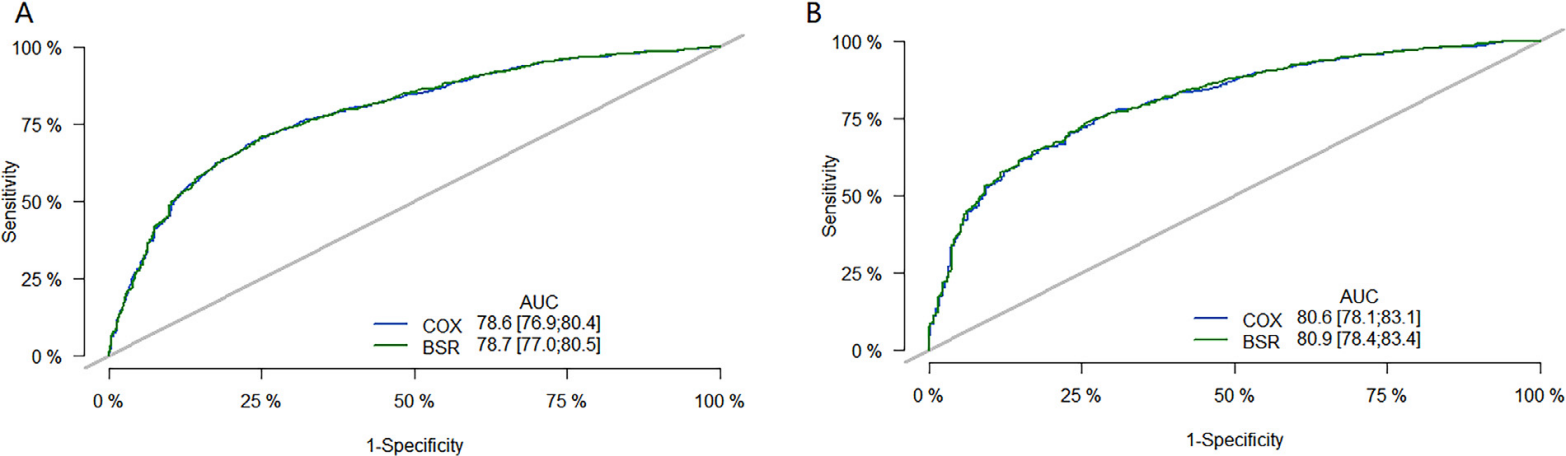
36-month ROC curve of models. (A) is the ROC curve of the model on the training set; (B) is the ROC curve of the model on the validation set. The green curve is the ROC curve of the BSR/LASSO model, and the gray is the ROC curve of the Cox single factor. The BSR/LASSO model performed better in both datasets (78.7 > 78.6; 80.9 > 80.6).

The performance of the model was then evaluated using ROC plots. Fig. 3 is the long-term (36 month) ROC curve of the model; A was the ROC curve of the model on the training set; B was the ROC curve of the model on the validation set. By comparing the AUC size, it was found that the BSR/LASSO model performs better in both datasets.

### Construction of the prognostic model

**Fig. 4:**
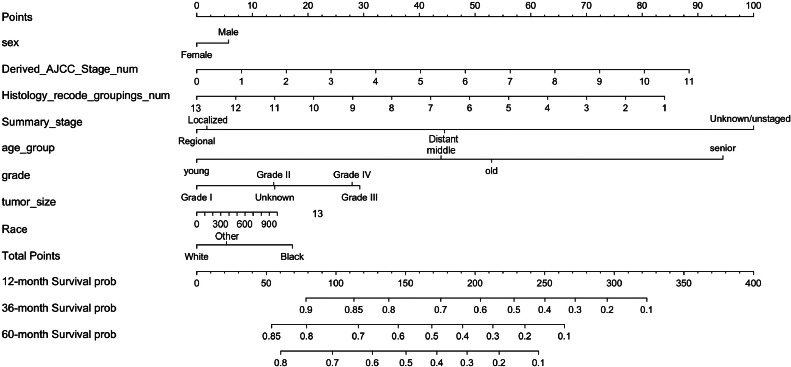
The Nomogram of EC patients. Points represents the single item score corresponding to each variable under different values, and Total Point represents the total score of the sum of the corresponding single item scores after all variable values. 12-month Survival probability, 24-month Survival prob, 36-month Survival prob represent 1-year, 3-year and 5-year survival probability, respectively. Each Total Points corresponds to 1-year, 3-year and 5-year survival rates.

In summary, eight variables were used as independent prognostic factors for EC, and a nomogram was constructed according to the prediction model, see Fig. 4. As can be seen from the figure, the tumor stage (Summary_Stage, Derived_AJCC_Stage_num) had the greatest impact on prognosis. It is undeniable that the AJCC cancer staging manual is still very important for predicting the survival of EC patients and guiding treatment. However, other social and demographic information and clinicopathological features should not be ignored, such as regional lymph node involvement, and distant metastasis, which have an impact on EC prognosis second only to tumor staging. The regional lymph node is closely related to tumor staging and is also malignant. The most common and most easily metastatic site of tumors, the enlargement or metastasis of regional lymph nodes indicates the prognosis of the tumor and also serves as a guide for the selection of surgical procedures.^31^ In addition, the number of lymph node metastases also guides the choice of chemotherapy regimens after surgery.^32^ It is recommended that patients with EC undergo periodic regional lymphatic examinations.

### Survival analysis

**Fig. 5:**
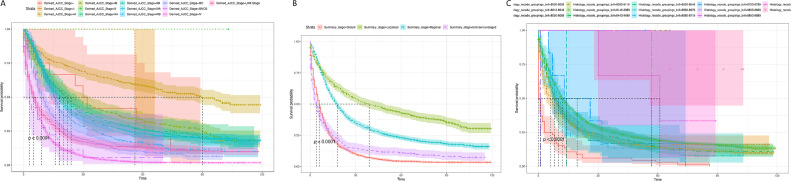
Kaplan Meier survival curve of EC patients.

Subsequently, the effects of tumor stage and regional lymph node involvement, and distant metastasis on patient survival were examined by Kaplan-Meier survival curves, as shown in Fig. 5. The results were consistent with the nomogram predictions. A is the Kaplan-Meier survival curve of EC patients at each stage in the AJCC stage; B is the Kaplan-Meier survival curve of EC patients at each stage in the Summary Stage; C is the different regional lymph node involvement, and distant metastasis Corresponding Kaplan-Meier survival curves for EC patients. Both A and B indicated that EC patients in the earliest stage had the highest survival rate; C revealed that lymph node metastasis patients had a lower survival rate, while distant metastasis patients had the lowest survival rate.

### Verification of model performance

**Fig. 6:**
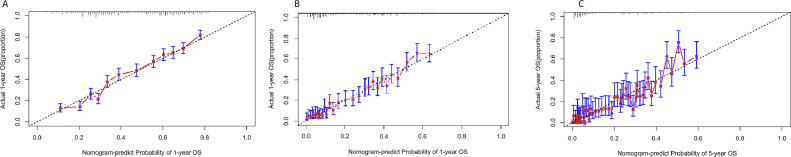
Calibration curve of the nomogram. The horizontal axis is the predicted event rate (Predicted risk), and the vertical axis is the observed actual event rate (Observed risk) with a range of 0 to 1. The dotted line on the diagonal is the reference line, that is, the prediction result perfectly matches the real result, and the red line is the fitting line, that is, the prediction of the model. The closer the two are, the better the calibration of the model.

**Fig. 7:**
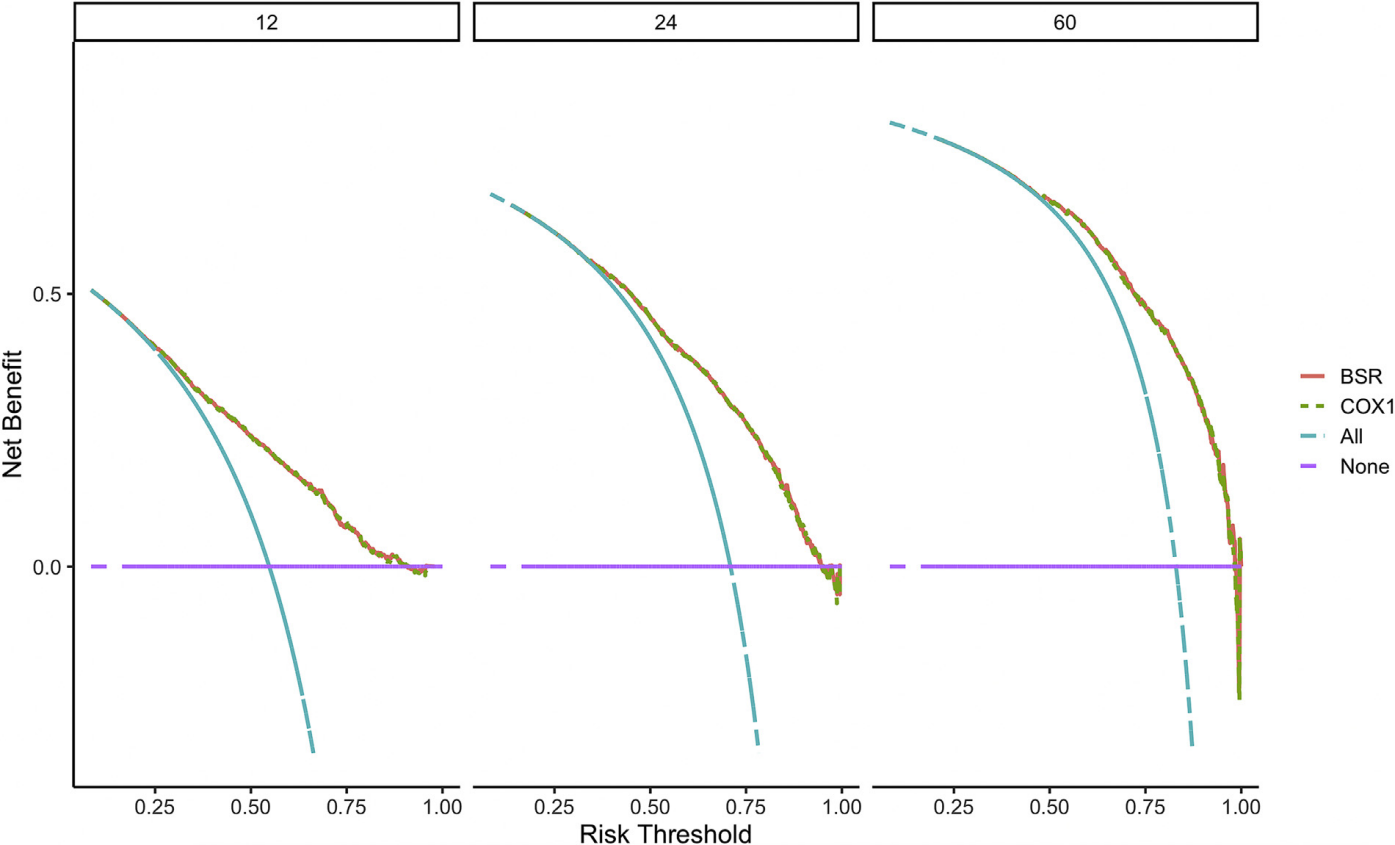
DCA curve of the nomogram. The horizontal axis of the DCA curve is the threshold probability, and the vertical axis is the net benefit. As the threshold probability increased, the net benefit of the model decreased (more precisely, the net benefit of intervention based on the model results decreased).

After the model was built, in order to evaluate the model and verify the difference between the survival rate predicted by the model and the actual, this paper drew the calibration curve of the model (Fig. 6). The effect of the prediction model was generally consistent with the actual survival situation, which further verified the distinguishing ability and calibration ability of the model. A, B, and C were calibration curves of the patient's 1-year, 3-year, and 5-year survival rates, respectively. The results showed that the model had a better prediction effect on the 1-year and 3-year survival rates of EC patients, and the 3-year effect was the best. Next, in order to evaluate the degree of patient benefit, a “threshold probability” was introduced using Decision Curve Analysis (DCA). Triggering medical intervention at the same threshold probability had high clinical utility. The decision analysis curve compares the net benefit of the intervention according to the model with the net benefit of the default approach (full and no intervention).^33^^,^^34^ Fig. 7 shows the 12-month, 24-month and 60-month Monthly decision analysis of the same model. As shown in the figure, for the trained Model, if the model was intervened according to the prediction results of the Model, except for the case where the threshold probability was small, the performance of the model was relatively good in the rest of the threshold probability cases, and the 60-month the highest net benefit.

## Discussion

In this study, a total of 5037 cases of EC patients with complete information were extracted from the SEER database, and differences in clinical and pathological factors affecting patient prognosis were explored. Subsequently, patients were randomly assigned to training and validation groups in a 7:3 ratio. Multiple independent risk factors were identified through multivariate and univariate analyses. These factors were further integrated into a nomogram to predict the 1-year, 3-year, and 5-year Overall Survival (OS) probabilities, and the high accuracy of this nomogram was demonstrated through internal and external validation. Compared to TNM staging, the 7^th^ edition AJCC staging, and SEER staging, this nomogram showed superior survival prediction capabilities, providing guidance for prognosis assessment for EC patients in terms of individualized tumor-specific survival predictions.

As indicated by the nomogram model in this study, gender has a minor impact on EC, consistent with findings from Zeng et al.'s research.^35^ Tumor staging has the greatest impact on EC, with more severe differentiation leading to poorer prognosis. As SEER staging increases, tumors progress, survival time shortens, and prognosis worsens, aligning with the trends identified by the nomogram model. Undeniably, the AJCC Cancer Staging Manual remains crucial for predicting the survival and guiding treatment of EC patients. Other factors such as regional lymph node involvement and distant metastasis have an impact on EC prognosis second only to tumor staging. Regional lymph nodes are the most susceptible sites for tumor metastasis, and their enlargement or metastasis indicates the tumor's prognosis and guides the choice of surgical approach.^36^ The number of lymph node metastases also guides the selection of postoperative chemotherapy regimens.^37^ Therefore, regular regional lymph node examination is recommended for EC patients. Nevertheless, unilateral AJCC staging does not satisfactorily predict prognosis, especially in patients with similar staging, due to confounding factors affecting EC prognosis. Therefore, compared to TNM staging, column-line diagrams are a simpler and more visual tool for estimating risk based on patient characteristics and are widely used in oncology and medical prognosis.^38^

To ensure the model's performance, this study employed calibration and Decision Curve Analysis (DCA) curves to validate the predicted survival rates against actual outcomes and assess clinical utility. The results show that the predicted 1-year and 3-year survival rates of EC patients are consistent with the actual situation, but there is a certain gap in the 5-year survival rate. Decision analysis showed that the model yielded higher 5-year survival in EC patients. Besides, this study has some limitations. Firstly, in terms of data acquisition, there were no suitable multiple data sources for external validation. Secondly, ESCC and EAC are the two main histological subtypes of EC with significant differences in epidemiology, tumor characteristics and genetic features.^39^ Therefore, the performance of column line plots in the two subtypes must be evaluated separately in subsequent studies. The SEER database did not include comprehensive treatment records, excluding treatment methods from the scope of this study. Furthermore, this retrospective study based on the SEER database requires further validation through prospective cohort studies to obtain sufficient evidence to verify the research results.

Despite these limitations, commonly used metrics such as the C-index, AUC, and calibration curves demonstrate the high accuracy of the nomogram. Compared to traditional staging, DCA suggests that the nomogram has better practicality.

## Conclusion

In summary, the nomogram model serves as an efficient individualized tool for predicting EC patient prognosis with better survival prediction ability, aiding clinicians in making informed treatment decisions.

Availability of data and materials

The datasets used and/or analyzed during the present study are available from the corresponding author upon reasonable request.

## Ethics statement

Not applicable. The present study did not require ethical board approval because it did not contain human or animal trials.

## Authors’ contributions

Xiying Cao designed the research study. Bingqun Wu and Shaoming Guo performed the research. Weixiang Zhong and Zuxiong Zhang provided help and advice on the experiments. Hui Li analyzed the data. Xiying Cao wrote the manuscript. Xiying Cao and Hui Li reviewed and edited the manuscript. All authors contributed to editorial changes in the manuscript. All authors read and approved the final manuscript.

## Funding

The Doctoral Initiation Fund Project from the First Affiliated Hospital of Gannan Medical University.

## Conflicts of interest

The authors declare no conflicts of interest.

## References

[1] Malhotra GK, Yanala U, Ravipati A, Follet M, Vijayakumar M, Are C. (2017). Global trends in esophageal cancer. J Surg Oncol.

[2] Layke JC, Lopez PP. (2006). Esophageal cancer: a review and update. Am Fam Physician.

[3] Short MW, Burgers K, Fry V. (2017). Esophageal cancer. Am Fam Physician.

[4] Abbas G, Krasna M. (2017). Overview of esophageal cancer. Ann Cardiothorac Surg.

[5] Uhlenhopp DJ, Then EO, Sunkara T, Gaduputi V. (2020). Epidemiology of esophageal cancer: update in global trends, etiology and risk factors. Clin J Gastroenterol.

[6] Vazquez-Sequeiros E, Wiersema MJ, Clain JE, Norton ID, Levy MJ, Romero Y (2003). Impact of lymph node staging on therapy of esophageal carcinoma. Gastroenterology.

[7] Llovet JM, Brú C, Bruix J. Prognosis of hepatocellular carcinoma: the BCLC staging classification Seminars in liver disease. © 1999 by Thieme Medical Publishers, Inc., 1999;19(03):329-38. 10.1055/s-2007-1007122 10518312

[8] Nagtegaal ID, Quirke P, Schmoll HJ. (2012). Has the new TNM classification for colorectal cancer improved care?. Nat Rev Clin Oncol.

[9] Massard G, Renaud S, Reeb J (2016). N2-IIIA non-small cell lung cancer: a plea for surgery!. J Thorac Dis.

[10] Su D, Zhou X, Chen Q, Jiang Y, Yang X, Zheng W (2015). Prognostic nomogram for thoracic esophageal squamous cell carcinoma after radical esophagectomy. PLoS One.

[11] Zheng Y, Fu S, He T, Yan Q, Di W, Wang J (2018). Predicting prognosis in resected esophageal squamous cell carcinoma using a clinical nomogram and recursive partitioning analysis. Eur J Surg Oncol.

[12] Veronesi U, Viale G, Rotmensz N, Goldhirsch A. (2006). Rethinking TNM: breast cancer TNM classification for treatment decision-making and research. Breast.

[13] Leung TWT, Tang AMY, Zee B, Lau WY, Lai PBS, Leung KL (2002). Construction of the Chinese University prognostic index for hepatocellular carcinoma and comparison with the TNM staging system, the okuda staging system, and the cancer of the liver italian program staging system: a study based on 926 patients. Cancer..

[14] Edge SB, Compton CC. (2010). The American Joint Committee on Cancer: the 7th edition of the AJCC cancer staging manual and the future of TNM. Ann Surg Oncol.

[15] Warner CL, Cockerell CJ. (2011). The new seventh edition American joint committee on cancer staging of cutaneous non-melanoma skin cancer. Am J Clin Dermatol.

[16] Hari DM, Leung AM, Lee J-H, Sim M-S, Vuong B, Chiu CG (2013). AJCC Cancer Staging Manual 7th edition criteria for colon cancer: do the complex modifications improve prognostic assessment?. J Am Coll Surg.

[17] Kattan MW, Reuter V, Motzer RJ, Katz J, Russo P. (2001). A postoperative prognostic nomogram for renal cell carcinoma. J Urol.

[18] Brennan MF, Kattan MW, Klimstra D, Conlon K. (2004). Prognostic nomogram for patients undergoing resection for adenocarcinoma of the pancreas. Ann Surg.

[19] Yin QH, Liu BZ, Xu MQ, Tao L, Wang K, Li F (2020). A Nomogram based on preoperative clinical bio-indicators to predict 5-year survivals for patients with gastric cancer after radical gastrectomy. Cancer Manag Res.

[20] Yan Y, Zhou Q, Zhang M, Liu H, Lin J, Liu Q (2020). Integrated nomograms for preoperative prediction of microvascular invasion and lymph node metastasis risk in hepatocellular carcinoma patients. Ann Surg Oncol.

[21] Liu J, Huang X, Yang W, Li C, Li Z, Zhang C (2020). Nomogram for predicting overall survival in stage II-III colorectal cancer. Cancer Med.

[22] Karakiewicz PI, Briganti A, Chun FK-H, Trinh Q-D, Perrotte P, Ficarra V (2007). Multi-institutional validation of a new renal cancer–specific survival nomogram. J Clin Oncol.

[23] Shariat SF, Margulis V, Lotan Y, Montorsi F, Karakiewicz PI. (2008). Nomograms for bladder cancer. Eur Urol.

[24] Cao J, Yuan P, Wang L, Wang Y, Ma H, Yuan X (2016). Clinical nomogram for predicting survival of esophageal cancer patients after esophagectomy. Sci Rep.

[25] Yu JB, Gross CP, Wilson LD, Smith BD. (2009). NCI SEER public-use data: applications and limitations in oncology research. Oncology (Williston Park).

[26] Arnal MJD, Arenas ÁF, Arbeloa ÁL. (2015). Esophageal cancer: Risk factors, screening and endoscopic treatment in Western and Eastern countries. World J Gastroenterol.

[27] Reeh M, Nentwich MF, von Loga K, Schade J, Uzunoglu FG, Koenig AM (2012). An attempt at validation of the Seventh edition of the classification by the International Union Against Cancer for esophageal carcinoma. Ann Thorac Surg.

[28] McCarthy CE, Field JK, Marcus MW. (2017). Age at menopause and hormone replacement therapy as risk factors for head and neck and oesophageal cancer. Oncol Rep.

[29] Wu S-G, Zhang W-W, Sun J-Y, Li F-Y, Lin Q (2018). He Z-Y. Patterns of distant metastasis between histological types in esophageal cancer. Front Oncol.

[30] Mountain CF, Dresler CM. (1997). Regional lymph node classification for lung cancer staging. Chest.

[31] Hu Y, Hu C, Zhang H, Ping Y, Chen L-Q (2010). How does the number of resected lymph nodes influence TNM staging and prognosis for esophageal carcinoma?. Ann Surg Oncol.

[32] Randolph GW, Duh QY, Heller KS, LiVolsi VA, Mandel SJ, Steward DL (2012). American Thyroid Association Surgical Affairs Committee's Taskforce on Thyroid Cancer Nodal Surgery. The prognostic significance of nodal metastases from papillary thyroid carcinoma can be stratified based on the size and number of metastatic lymph nodes, as well as the presence of extranodal extension. Thyroid..

[33] Vickers AJ, Elkin EB. (2006). Decision curve analysis: a novel method for evaluating prediction models. Med Decis Making.

[34] Vickers AJ, van Calster B, Steyerberg EW. (2019). A simple, step-by-step guide to interpreting decision curve analysis. Diagn Progn Res.

[35] Zeng Y, Ruan W, Liu J, Liang W, He J, Cui F (2018). Esophageal cancer in patients under 50: a SEER analysis. J Thorac Dis.

[36] Eloubeidi MA, Desmond R, Arguedas MR, Reed CE, Wilcox CM. (2002). Prognostic factors for the survival of patients with esophageal carcinoma in the US: the importance of tumor length and lymph node status. Cancer.

[37] Greenstein AJ, Litle VR, Swanson SJ, Divino CM, Packer A, Wisnivesky JP. (2008). Prognostic significance of the number of lymph node metastases in esophageal cancer. J Am Coll Surg.

[38] Balachandran VP, Gonen M, Smith JJ, DeMatteo RP. (2015). Nomograms in oncology: more than meets the eye. Lancet Oncol.

[39] Zhang X, Wang Y, Meng L. (2022). Comparative genomic analysis of esophageal squamous cell carcinoma and adenocarcinoma: New opportunities towards molecularly targeted therapy. Acta Pharm Sin B.

